# Anterior cruciate ligament injury should not be considered a contraindication for medial unicompartmental knee arthroplasty: Finite element analysis

**DOI:** 10.1371/journal.pone.0299649

**Published:** 2024-03-12

**Authors:** Deyan Ou, Yongqing Ye, Jingwei Pan, Yu Huang, Haisheng Kuang, Shilin Tang, Richao Huang, Yongxin Mo, Shixin Pan

**Affiliations:** 1 Department of Limb and Joint Ward, Wuzhou Red Cross Hospital, Wuzhou, Guangxi Province, China; 2 Department of Medical Imaging Department, Wuzhou Red Cross Hospital, Wuzhou, Guangxi Province, China; 3 Department of Spine Ward, Wuzhou Red Cross Hospital, Wuzhou, Guangxi Province, China; Ningbo University, CHINA

## Abstract

**Purpose:**

The research objective of this study is to use finite element analysis to investigate the impact of anterior cruciate ligament (ACL) injury on medial unicompartmental knee arthroplasty (UKA) and explore whether patients with ACL injuries can undergo UKA.

**Methods:**

Based on the morphology of the ACL, models of ACL with diameters ranging from 1 to 10mm are created. Finite element models of UKA include ACL absence and ACLs with different diameters. After creating a complete finite element model and validating it, four different types of loads are applied to the knee joint. Statistical analysis is conducted to assess the stress variations in the knee joint structure.

**Results:**

A total of 11 finite element models of UKA were established. Regarding the stress on the ACL, as the diameter of the ACL increased, when a vertical load of 750N was applied to the femur, combined with an anterior tibial load of 105N, the stress on the ACL increased from 2.61 MPa to 4.62 MPa, representing a 77.05% increase. Regarding the equivalent stress on the polyethylene gasket, a notable high stress change was observed. The stress on the gasket remained between 12.68 MPa and 14.33 MPa in all models. the stress on the gasket demonstrated a decreasing trend. The equivalent stress in the lateral meniscus and lateral femoral cartilage decreases, reducing from the maximum stress of 4.71 MPa to 2.61 MPa, with a mean value of 3.73 MPa. This represents a reduction of 44.72%, and the statistical significance is (P < 0.05). However, under the other three loads, there was no significant statistical significance (P > 0.05).

**Conclusion:**

This study suggests that the integrity of the ACL plays a protective role in performing medial UKA. However, this protective effect is limited when performing medial UKA. When the knee joint only has varying degrees of ACL injury, even ACL rupture, and the remaining structures of the knee joint are intact with anterior-posterior stability in the knee joint, it should not be considered a contraindication for medial UKA.

## 1. Introduction

Knee osteoarthritis (KOA) is the most common type of arthritis affecting the joints and is the most prevalent degenerative joint disease in the elderly. Approximately one-third of patients are affected unilaterally, resulting in irregular chronic pain in the affected compartment. These changes significantly decrease their quality of life [1–3]. Medial UKA is often used for patients with medial compartment osteoarthritis (MOA). Ideally, suitable candidates for medial UKA are MOA patients who have intact ACL. Due to its minimally invasive nature and preservation of proprioception, along with excellent surgical outcomes, the indications for medial UKA are expanding to include patients with anterior cruciate ligament deficiency (ACLD) [4–7]. In a normal knee joint, the ACL plays a crucial role in knee joint movement and maintaining stability. The traditional belief is that in knee joints without the ACL, it can result in anterior tibial translation and abnormal shear forces on the posterior medial side of the knee joint. This can worsen knee joint degeneration and eventually lead to the development of osteoarthritis [8–10]. In a series of UKA surgeries on 103 knee joints conducted by Goodfellow et al., an increased rate of joint replacement failure was reported to be closely related to the ACLD. ACLD has traditionally been considered a contraindication for UKA. However, recent research has challenged previous conclusions and showed that UKA can successfully treat ACLD knee joints. Studies by Boissonneault et al. and Kikuchi et al. have indicated that the component survival rates in recipients of UKA with intact and absent ACL were similar during an average follow-up of 5 years [4, 11]. Another research report that there is no difference in revision rates and functional outcomes between the ACLD group and the ACL-intact group [12].

The finite element analysis method is frequently utilized in the biomechanical research of knee joints. Daszkiewicz, for instance, simulated degenerative changes in the medial knee joint osteoarthritis (OA) model by introducing non-uniform reduction in articular cartilage thickness in specific regions and reducing material parameters of cartilage and meniscus [13]. Wen conducted a study by establishing a finite element model of the knee joint to investigate the impact of lower limb alignment and tibial component inclination on the biomechanics of the lateral compartment in unicompartmental knee arthroplasty [14]. Kwon, Ma, and others conducted a finite element analysis of the knee joint, separately investigating the importance of preserving the joint line in unicompartmental knee replacement surgery and the biomechanical effects of femoral components in different coronal positions during medial unicompartmental knee replacement surgery [15, 16].The impact of ACLD on UKA remains controversial and the related biomechanical analysis of the knee joint is also lacking, and there is currently no consensus on whether the ACL is a necessary prerequisite for successful surgery. More research is needed to compare the various outcomes of ACLI and ACLD cases. The objective of this study is to employ finite element analysis to establish finite element models of knee joints with different degrees of ACL tears and analyze the impact of these tears on the biomechanical performance of the knee joint after UKA.

## 2. Materials and methods

### 2.1 Establishment of a complete and normal knee joint finite element model

**Fig 1:**
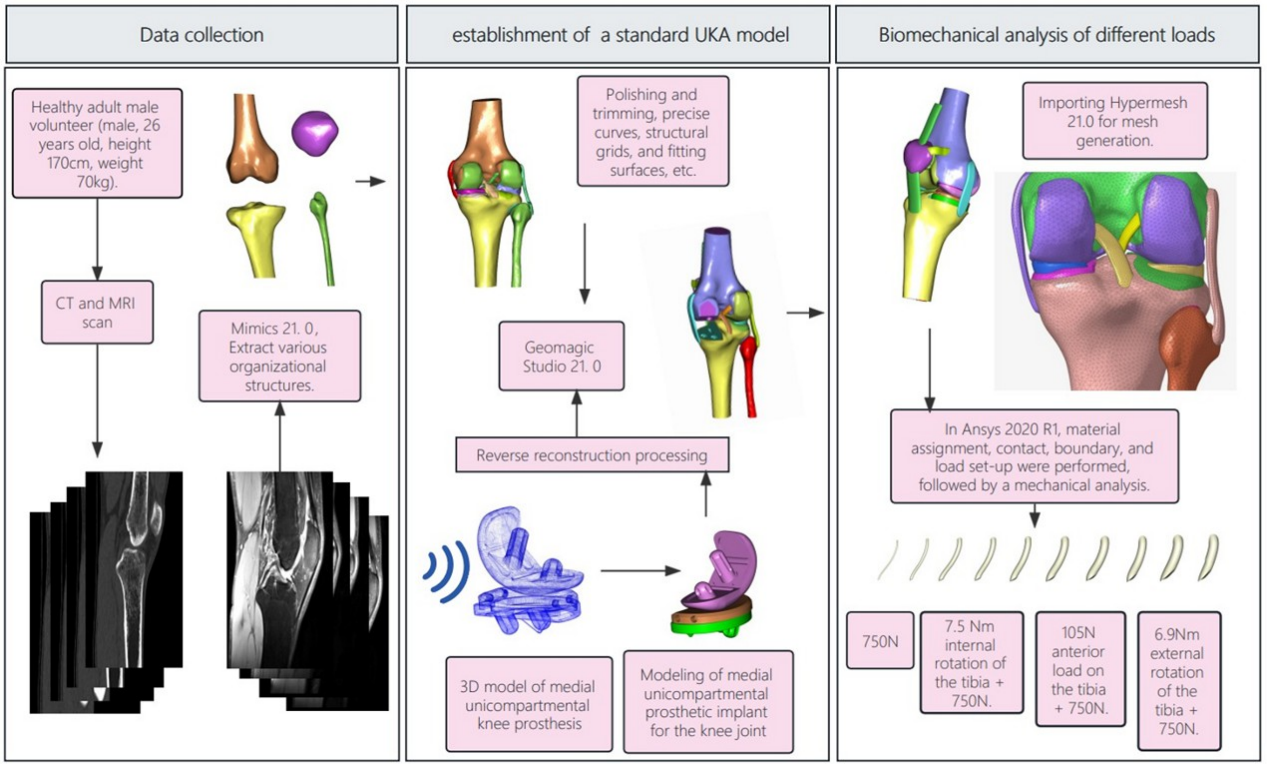
The flowchart of the entire research process: Data collectin, establishment of standard UKA model, biomechanical analysis of different loads.

Select a healthy adult male volunteer (male, 26 years old, height 170cm, weight 70kg) for knee joint CT and MR imaging. With the consent of the subject, the scanned images will be used for this study. The study will be conducted in a supine position with both lower limbs extended at 0°. Spiral CT scanning will be performed using the GE RevolutionCT device at the Medical Imaging Center of the Red Cross Hospital in Wuzhou City. The scanning range will cover the distal femur to the proximal tibia and fibula. The slice thickness and interspace will be 0.625mm, and the reconstruction thickness and spacing will also be 0.625mm. The tube voltage will be 120kV, and the current will be 240mA. The original data will be exported and archived in DICOM format. The images will be imported into the medical processing software Mimics 21.0 (Materialise NV, Leuven, Belgium). The knee joint model will be obtained by extracting the images through successive two-dimensional segmentation based on the structure of the knee joint components. The model will be exported in STL format and imported into the reverse engineering software Geomagic Studio 21.0 (Geomagic, North Carolina, USA). Various parts of the model will be polished and refined, including precise surface, structural lattice, and fitted surface processing. NURBS surfaces will be generated, and the model will be exported in STP format and imported into the finite element pre-processing software Hypermesh 21.0 (Altain Corporation, USA). The three-dimensional model will be meshed to simulate knee joint biomechanics more realistically. The mesh will be further refined at the ligament attachment sites, meniscus, and cartilage, with an average boundary of 0.8mm for the meniscus mesh, 1.0mm for the cartilage mesh, and 1.5mm for the bone mesh(Table 1). The meniscus, cartilage, and ligaments will have a shared node. Contact relations will be established between the articular cartilage and meniscus, and a total of 37 contact relations will be established between bone, cartilage, and ligaments. The cortical and trabecular bones will be set as bound. The cartilage will be bound to the bone surface, and there will be frictional contact between the cartilage and between the meniscus and cartilage, with a friction coefficient of 0.1. This will establish a intact finite element model of the knee joint, including the femur, tibia, fibula, femoral cartilage, tibial cartilage, and meniscus (Fig 1).

**Table 1:** Elements and nodes of different items.

Items	Elements	Nodes	Items	Elements	Nodes
Lateral meniscus	5492	1674	Tibia-cortical bone	36613	12279
Patella-cartilage	4820	1497	Tibia-trabecular bone	63842	14467
Patella-cortical bone	14473	4678	LCL	11611	3036
Patella-trabecular bone	20499	4728	MCL	9823	2665
Fibula-articular cartilage	1507	538	PCL	4954	1315
Fibula-cortical bone	13797	4520	Patellar tendon.	11713	2950
Fibula-trabecular bone	9974	2609	1	5949	1839
Femur-lateral cartilage	13646	4430	2	5343	1511
Femur-prosthesis	35870	8603	3	7351	1940
Femur-bone cement	15601	4973	4	9240	2323
Femur-cortical bone	37812	12851	5	9546	2384
Femur-trabecular bone	69174	15522	6	12530	3029
Tibia-fibula articular cartilage	2380	858	7	11736	2832
Tibia-lateral articular cartilage	6312	2014	8	12245	2945
Tibia-prosthesis	20583	5735	9	12026	2885
Gasket	23300	5626	10	12086	2889
Tibia-bone cement	11400	3886			

### 2.2 Establishment of UKA finite element model and material assignment

A three-dimensional model of the medial UKA is created, which includes components such as the femoral component, tibial component, and polyethylene insert. The model utilizes the Zimmer (Zimmer Inc., Warsaw, IN, USA) fixed-bearing medial unicompartmental knee prosthesis. Each component is scanned using a 3D scanner and then imported into Geomagic Studio 21.0 for further reverse reconstruction processing. This process generates the femoral component, PE gasket, and tibial platform tray. After reconstruction, the components are assembled into the previously established knee joint model based on the standard unicompartmental replacement surgical procedure. After assembly, a 1mm gap is created between the mobile component and the femur and tibia, and it is automatically filled with bone cement to a thickness of 1mm. Once the assembly is complete, the model is imported into Hypermesh 21.0 for meshing. Referring to the method of establishing a complete and normal knee joint finite element model, a finite element model for UKA surgery is established. Once the complete and normal knee joint finite element model and UKA finite element model are established, they are imported into Ansys 2020R1 for material assignment in inp format. The ligament models were assumed to be isotropic and hyperelastic materials in order to represent their nonlinear stress-strain relationships. These relationships were described using an incompressible Neo-Hookean behavior with an energy density function of Ψ = C_1_ × (I_1_−3), where C_1_ represents the initial shear modulus and I1 represents the first modified invariant of the right Cauchy-Green strain tensor. The specific C_1_ values for the lateral collateral ligament, medial collateral ligament, anterior cruciate ligament, and posterior cruciate ligament were determined to be 6.06, 6.43, 5.83, and 6.06 MPa, respectively [17, 18]. The meniscus is defined as a transversely isotropic material, while the rest of the tissue structures are defined as linear elastic isotropic materials. The parameters for these tissue structures have been assigned based on previous experimental results. The elastic modulus and Poisson’s ratio of the tissue structures fall within a reasonable range and have been verified [16, 19, 20] (Table 2).

**Table 2:** Material properties of different items of knee.

Items	Young’s modulus	Poisson’s ratio
Cortical bone	17,000	0.3
Cobalt-chromium-molybde-num alloy	210,000	0.29
Methacrylate	1940	0.4
Cancellous bone	350	0.25
Menisci	27.5	0.33
Cartilage	15	0.46
Polyethylene(PE)	850	0.4

### 2.3 Load and boundary conditions and statistical analysis

**Fig 2:**
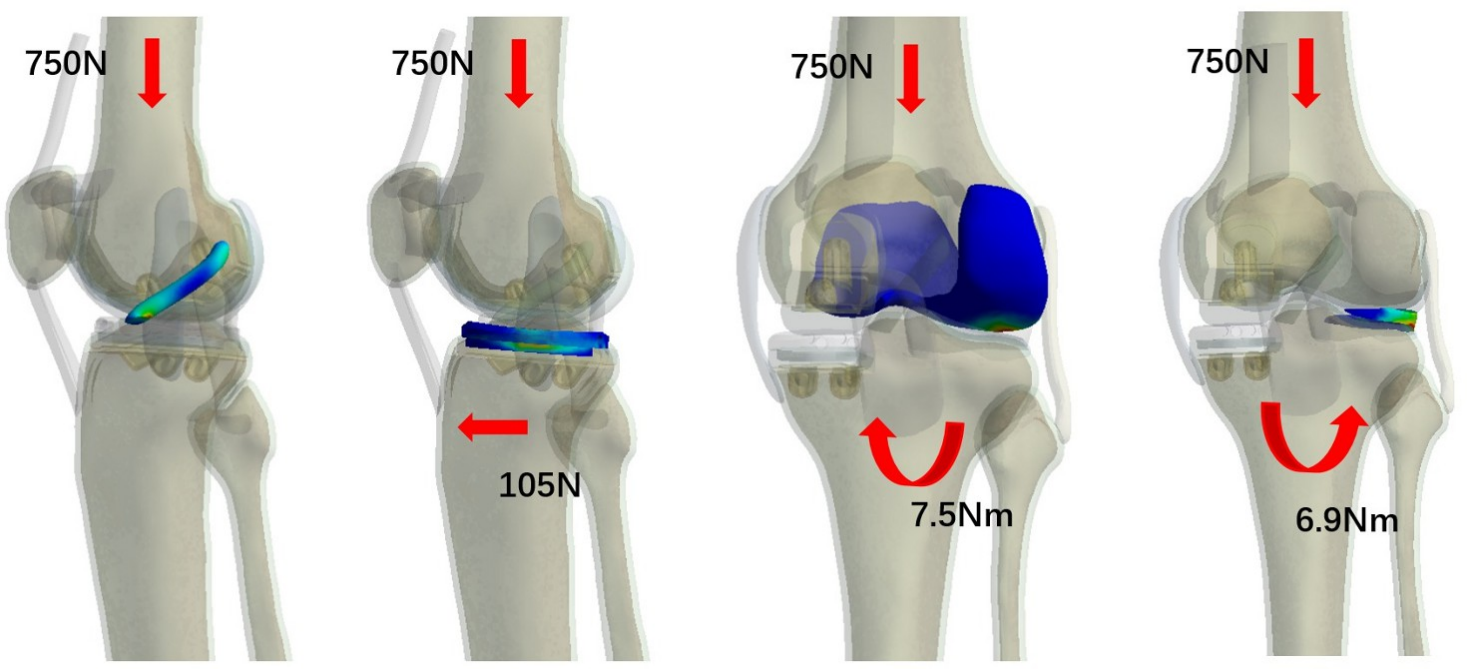
750N of vertical load on the femur; 750N of vertical load on the femur, combined with 105N of anterior tibial load; 750N of vertical load on the femur, combined with 7.5Nm of tibial varus load; 750N of vertical load on the femur, combined with 6.9Nm of tibial internal rotation load.

In addition to the original coordinate system, a local coordinate system is established on the femur to simulate the relative motion between the femur and tibia. The origin of the local coordinate system is set at the intersection of the line connecting the far ends of the medial and lateral condyles of the femur and the midline of the tibia. The X-axis (anteroposterior direction) represents the coronal plane axis of the femur, the Y-axis (superior-inferior axis) represents the mechanical axis of the femur, and the Z-axis (proximal-distal axis) is perpendicular to the X-axis and Y-axis. Therefore, the relative motion of the femur and tibia can be performed as follows: flexion-extension around the X-axis, internal-external rotation around the Y-axis, and abduction-adduction around the Z-axis. During this relative motion, the tibia and fibula remain fixed, while the femur moves relative to the tibia and exerts an axial load along the Y-axis and an anterior-posterior load along the Z-axis. By fixing the tibia and fibula with XYZ and -X-Y-Z six degrees of freedom at the bottom, selecting the femur as the solid entity, setting remote points and remote forces, coupling all nodes on the femur surface, selecting the midline of the medial and lateral condyles of the femur as the remote point, and setting the remote force direction downward, several loading conditions are simulated on the intact knee joint, including: ① Applying a longitudinal load of 750 N on the femur; ② Applying a longitudinal load of 750 N on the femur and an anterior-posterior load (105 N, 15% body weight); ③ Applying a longitudinal load of 750 N on the femur and an internal rotation moment of 7.5 Nm (1.1% body weight) to simulate knee internal rotation; ④ Applying a longitudinal load of 750 N on the femur and an internal rotation moment of 6.9 Nm (1% body weight) to simulate knee internal rotation [14, 19, 20] (Fig 2).Using Ansys software, four loading modes were applied to a three-dimensional finite element model of the knee joint to simulate the stress distribution in the extended position. This allowed for the calculation of peak equivalent stresses in the lateral meniscus, anterior cruciate ligament, and medial gasket, as well as the generation of stress contour maps. Comparisons were made between different groups. The diameter of the anterior cruciate ligament was systematically varied for each loading condition, and the four loading conditions were repeated. This was done to test the accuracy of the model analysis, with the loading conditions set to match the loading protocols used in biomechanical experiments on the same knee joint sample. We will collect the stress distribution data for the lateral meniscus, lateral femoral cartilage, polyethylene insert, and anterior cruciate ligament. We will statistically analyze their stress distributions, using a one-sample t-test, and consider a p-value less than 0.05 as statistically significant.

### 2.4 Ethics statement

This research has obtained approval from the Ethics Committee of Wuzhou Red Cross Hospital, conducted in accordance with the Helsinki Convention and in compliance with legislations. Written informed consent was obtained prior to the inclusion of participant in the study.

## 3. Results

### 3.1 Knee joint model validation

**Fig 3:**
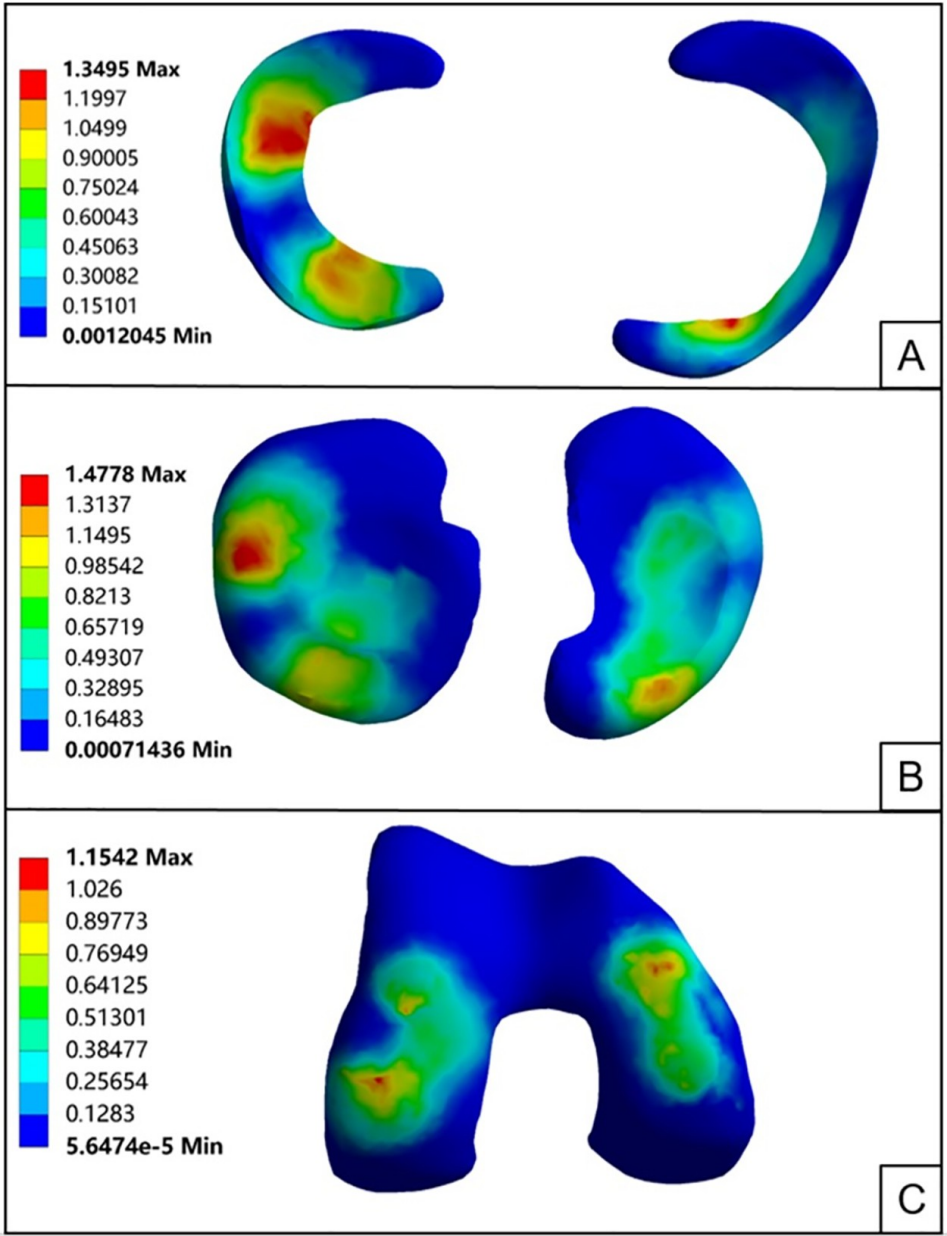
Validation of stress structure of knee joint: A: Equivalent stress of medial and lateral menisci; B: Equivalent stress of medial and lateral tibial cartilage; C: Equivalent stress of femoral cartilage.

The knee joint finite element model is subjected to loads and boundary conditions. The tibia is fixed at the bottom of the fibula in XYZ and -X-Y-Z directions to restrict its six degrees of freedom. A longitudinal load of 1000N is applied on the femur to simulate the load. Similarly, a forward pushing force of 134N is applied on the front side of the femur. With these inputs, you can calculate the contact pressure and displacement of the femur in the model [14, 21]. The knee joint finite element model consists of the femur, tibia, fibula, meniscus, articular cartilage of the femur, articular cartilage of the tibia, medial and lateral collateral ligaments, and anterior and posterior cruciate ligaments. It simulates the standing weight-bearing condition of a human body and analyzes the stress distribution in the meniscus, femoral articular cartilage, and tibial articular cartilage under a load of 1,000N. By comparing the calculated results in this study with previous research, it can be observed that the contact stress of the medial and lateral meniscus is 1.35 MPa and 1.33 MPa, respectively. This observation is consistent with the trends observed in previous studies under the same load conditions [22]. The contact stress of the medial and lateral tibial cartilage is 1.48 MPa and 1.28 MPa, respectively, while the contact stress of the femoral cartilage is 1.15 MPa. These results are very similar to the previous research findings [22–25]. Under a loading force of 134N applied from posterior to anterior, the average anterior displacement of the tibia is reported to be 4.53mm, which is consistent with previous studies [20] (Fig 3).

### 3.2 Equivalent stress variation of the ACL

**Fig 4:**
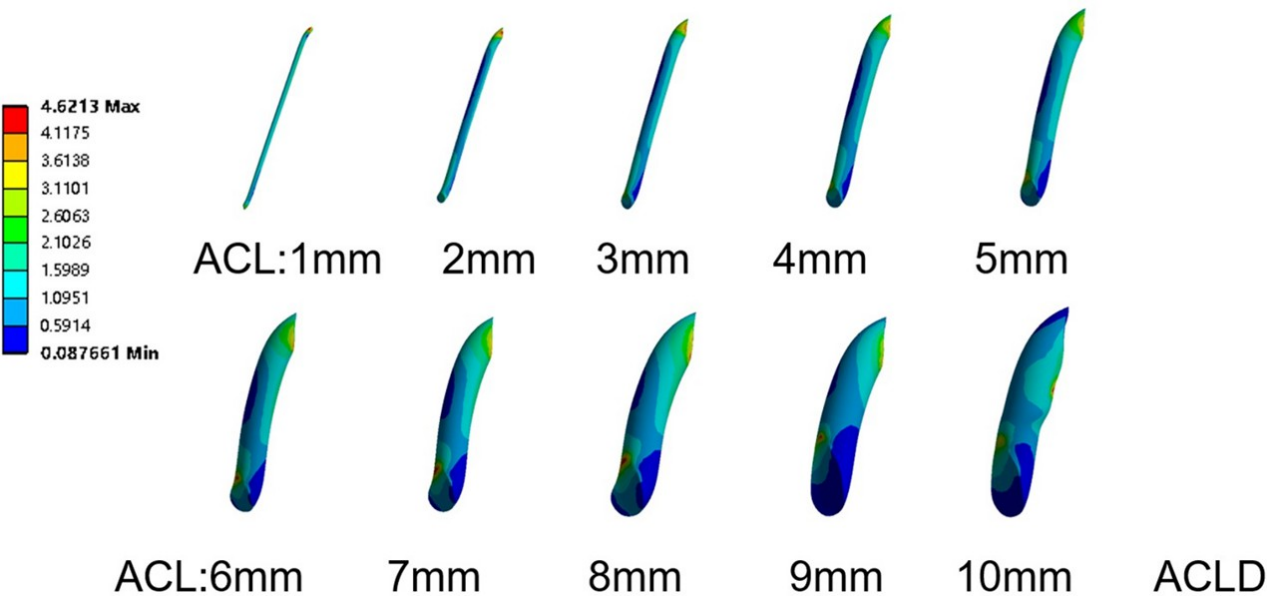
The equivalent stress distribution contour maps of the anterior cruciate ligament under a vertical load of 750 N on the femur and an anterior load of 105 N on the tibia.

Four different loads were applied to a total of 11 finite element models of knee joints, including knee joint models with ACL of diameters ranging from 1mm to 10mm, as well as knee joint models without ACL. These loads included vertical load on the femur, vertical load on the femur combined with anterior tibial load, vertical load on the femur combined with varus load on the tibia, and vertical load on the femur combined with internal rotation load on the tibia.Under the combination of vertical load and anterior tibial load, the stress on the ACL increased from 2.61 MPa to 4.62 MPa, with a 77.05% increase. This increase was statistically significant (P<0.05), and the equivalent stress reached its peak when the diameter of the ACL was 10mm. In the other three load conditions, the stress on the ACL remained relatively small, showing an overall increasing trend but without significant changes. The increase in stress was 0.32304 MPa, 0.32325 MPa, and 0.32322 MPa for the three respective loads. There was no significant difference in stress under the three rotational loads, and the statistical significance was not found (P>0.05) (Fig 4).

### 3.3 Equivalent stress variation of the polyethylene gasket

**Fig 5:**
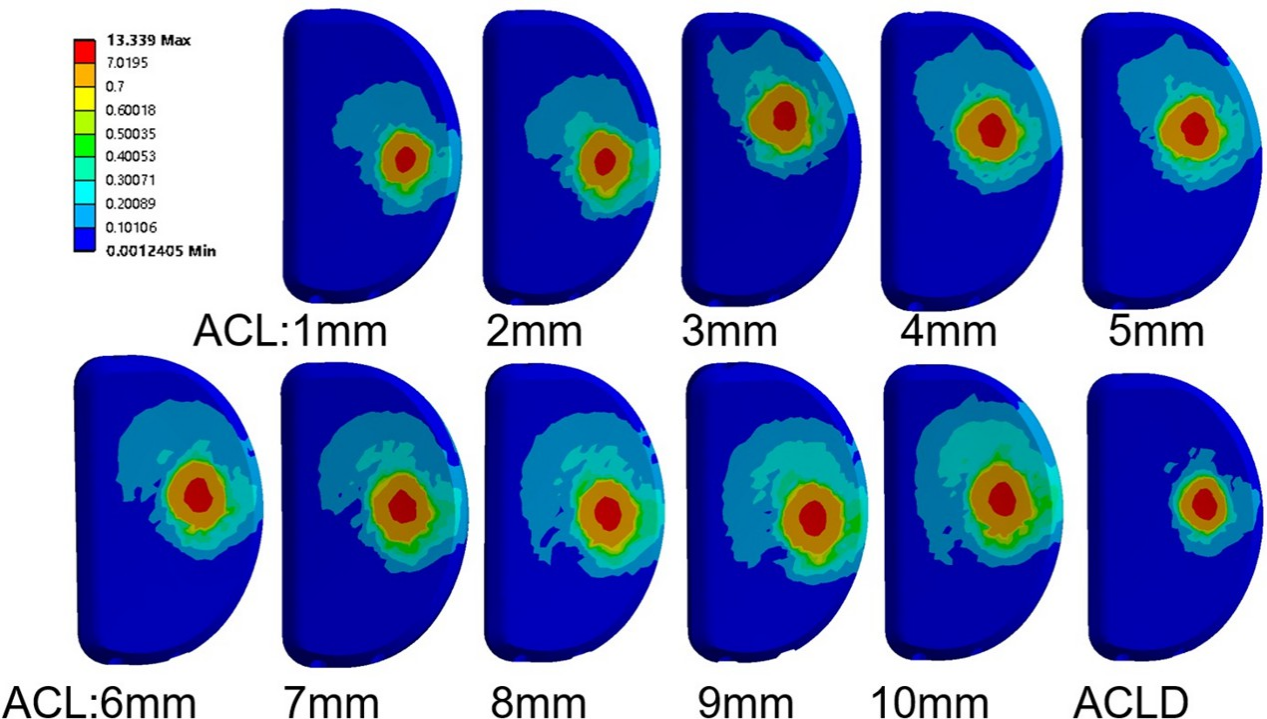
The stress distribution contour maps of the polyethylene gasket, under a vertical load of 750 N on the femur and an anterior load of 105 N on the tibia.

Polyethylene gasket shows a significant change in equivalent stress, with all results ranging from 12.68 MPa to 14.33 MPa, even though the ALC shares a part of the load. With an increase in the diameter of the ACL, the stress on the gasket tends to decrease. Among the models with a 6mm diameter ACL, the polyethylene gasket exhibits the lowest equivalent stress at 12.68 MPa. It shows a noticeable decrease in stress under the combination of vertical load and anterior tibial load, with a decrease of only 12.98%. This decrease is statistically significant (P<0.05). However, under the other three loads, there is no significant fluctuation in the equivalent stress of the polyethylene gasket, with average equivalent stresses of 14.11 MPa, 14.17 MPa and 14.10 MPa respectively. The statistical significance is not meaningful (Fig 5).

### 3.4 Equivalent stress variation in the lateral meniscus and lateral femoral cartilage

**Fig 6:**
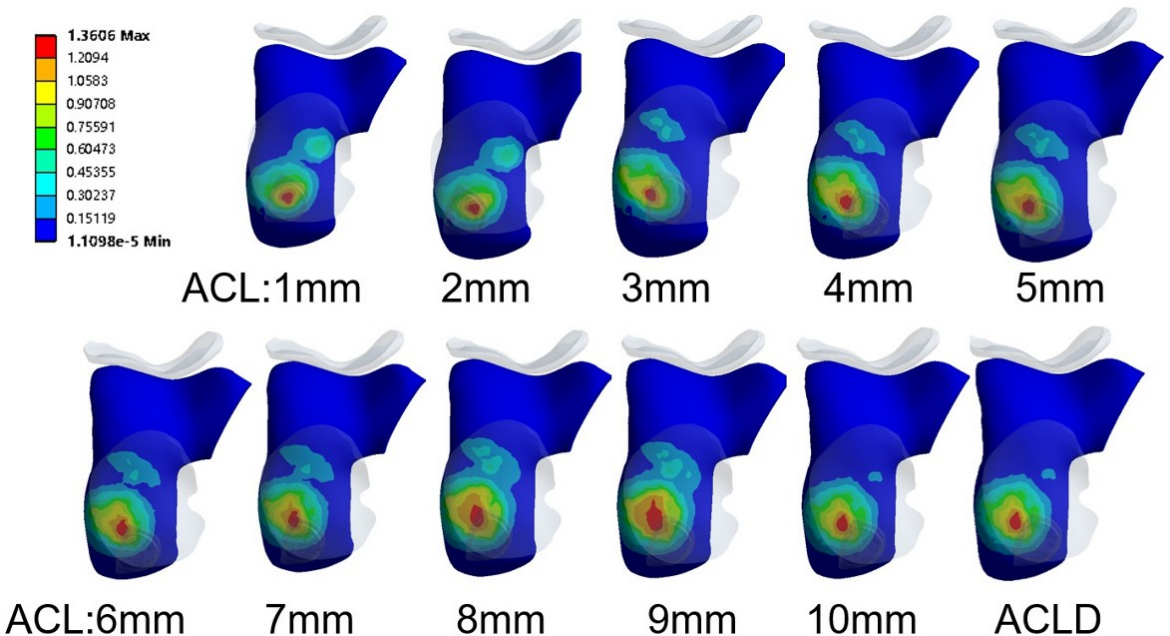
The stress distribution contour maps of the lateral femoral cartilage under a vertical load of 750 N on the femur and an anterior load of 105 N on the tibia.

With the increase in the diameter of the ACL, the lateral meniscus is protected during vertical loading with anterior tibial load combination, as evidenced by a significant decrease in stress. The maximum stress decreased from 4.72 MPa to 2.61 MPa, with an average of 3.73 MPa, resulting in a reduction of 44.72%. This reduction is statistically significant (P<0.05). In the other three loading conditions, there were no significant fluctuations in the equivalent stress of the polyethylene spacer, with average stresses of 1.211 MPa, 1.212 MPa, and 1.207 MPa, respectively. There was no statistically significant difference. Similarly, the stress changes in the lateral femoral cartilage exhibit a similar pattern to the lateral meniscus. The increase in the diameter of the ACL also reduces the equivalent stress in the femoral cartilage, decreasing from the maximum stress of 1.55 MPa to 1.17 MPa, with an average of 1.32 MPa, resulting in a reduction of 24.30%. This reduction is statistically significant (P<0.05). In the other three loading conditions, there were no noticeable fluctuations in the equivalent stress in the lateral femoral cartilage, with average stresses of 0.742 MPa, 0.743 MPa, and 0.743 MPa, respectively. There was no statistically significant difference (Fig 6).

## 4. Disscusion

Based on the validation of the model’s effectiveness, this study has constructed a new three-dimensional finite element model of a normal knee joint. Drawing from the advanced Zimmer medial unicompartmental knee prosthesis, a standard model of the knee joint after unilateral UKA was established. Additionally, different diameters of the ACL were simulated to mimic various degrees of tear. This was done to conduct an in-depth study on the impact of ACL injure on the stress distribution of the implanted unicompartmental knee prosthesis.

This includ all anatomical structures of the knee joint. For the challenging simulation of ligament structures, a hyperelastic rubber material was used, which better mimics the physiological structure of the knee joint compared to linear or spring ligaments. This approach allows for a more accurate representation of the biomechanical changes in the knee joint. Considering the anatomical position of the ACL, the shape of its insertion points, the cross-sectional area, and the length-to-width ratio in the middle, this design plan can be effectively used to investigate the impact of different degrees of ACL tears on medial UKA [18, 26]. In order to better understand the mechanical role of the ACL in stabilizing the knee joint, the simulation also considers the worst-case scenario of tensioning the ACL. In this situation, the functionality of the ACL can be thoroughly examined and its contribution to knee joint stability can be assessed more effectively [27, 28].

In this study, we believe that the integrity of the ACL plays a certain protective role in performing medial unicompartmental knee replacement surgery. However, it is not absolutely necessary, and knee joints with anterior cruciate ligament injury can still undergo knee replacement surgery. The ACL plays a role in limiting anterior translation of the tibia. As the diameter of the ACL increases, its stress increases, contributing to a portion of the load. It also partially reduces the stress on the lateral meniscus, lateral cartilage, and polyethylene insert, although this reduction is not significant. When applying internal and external rotational torques to the tibia, whether it’s the ACL, lateral meniscus, lateral cartilage, or polyethylene insert, there is no apparent increase in stress. However, drawing conclusions based solely on the results of this study is crude. We established a complete knee joint model, and in the structural stability of the knee joint, in addition to the ACL, there are intact posterior cruciate ligaments, medial and lateral collateral ligaments, patellar tendon, and quadriceps femoris, providing compensatory front-to-back stability for the knee joint [29–31]. In other words, even when there is isolated anterior cruciate ligament injury or tearing, and the accessory ligaments and structures of the knee joint are normal, the joint remains stable, and medial unicompartmental knee replacement surgery can still be performed.

With an increase in the diameter of the ACL, the stress gradually decreases under the vertical load combination with tibial anterior load. The overall reduction is approximately 1.6 MPa. This finding aligns with the research conducted by Peña et al., where they observed similar results. In their study, under a combined load of 1150 N vertical load and 134 N tibial anterior load, the equivalent stress fluctuation in the intact knee meniscus ranged from 3–6 MPa, which was slightly higher than the results obtained from this experiment. This difference can be attributed to the larger load used in their study [20]. Under three other loads, the changes in stress on the lateral meniscus and cartilage remain at a lower level with minimal fluctuations. The maximum and minimum stress on the lateral cartilage are only 0.74 MPa and 0.73 MPa, respectively. The maximum and minimum stress on the lateral meniscus are only 1.21 MPa and 1.19 MPa, respectively. This can be primarily attributed to the presence of the intact medial collateral ligament and lateral collateral ligament, which provide constraints on the external rotation and anterior-posterior translation of the knee joint, thus reducing the stress changes that occur in ACLD conditions. Previous research has also indicated this phenomenon [20, 31]. In a study by Thomas et al., they conducted a kinematic analysis comparing UKA in ACLI knees and UKA in ACLD knees. They used mobile fluoroscopy and tracked the knee joints of 10 patients who underwent conventional UKA and 8 patients with ACLD. Both groups showed similar motion waveforms. The researchers concluded that a fixed-bearing UKA with a 5–8° posterior tibial slope adjustment can partially compensate for the lack of ACL function and can be a feasible treatment option for ACLD patients. Furthermore, it can maintain a higher average survival period in their study [32–34]. These findings also explain why the contact structure stress in the knee joint does not show significant changes when the ACL is missing but the other knee ligaments are intact under rotational loads.

However, in clinical practical application, there are many factors that can lead to different outcomes, such as improvements in prosthesis design, proficiency and advancements in surgical techniques, and patient selection [35–37]. In clinical practice, apart from ACL rupture due to trauma, most cases of ACLD result from long-term chronic impacts and wear. Osteophyte formation is often present in the posterior part of the knee joint, and the joint capsule of the knee joint may also thicken accordingly. This contributes to the stability of the knee joint, and drawer tests as well as Lachman tests on these patients are often negative, indicating that their knee joints do not have functional anterior or posterior instability [38, 39]. In a study of patients with KOA, the knee joints of osteoarthritis patients are often more stable than those of normal knee joints. This is due to the combined effects of ligament contraction and the pressure exerted by osteophytes on ligaments and other capsular structures [40]. Later, Dayal and colleagues reported similar results, indicating that as the severity of knee joint osteoarthritis increases, the knee joint becomes relatively more stable anteriorly and posteriorly [41]. Therefore, the generation of osteophytes and soft tissue contraction can serve as a compensatory mechanism to stabilize the knee joint in the absence of the ACL. Performing medial UKA surgery is feasible for such patients.

This study has some limitations: (1) The model assumes that the ACL is a uniformly increasing diameter elliptical body. In actual cases of ACL injury, the damage to the ACL is not so regular. However, the method used in this study ensured that the size of the ACL was the only variable in drawing conclusions. This is one advantage of using finite element analysis over cadaver or in vivo studies. Strict control of variables allowed for the exclusion of individual differences and eliminated any influence on the studied characteristics. (2) All solid elements were set as isotropic material properties, which may not accurately reflect the material properties of all elements. However, the primary function of the ACL is to withstand tension, and the conditions used in this study adequately simulate physiological loading, specifically stretching the ACL. The isotropic characteristics assigned to the ligament are not expected to affect the main findings of this study. (3) The finite element model was simplified compared to the complex human joint, and the model’s geometry was based on a single sample. However, finite element analysis allows for variable changes without damaging samples, and when other factors are kept constant, variables can be strictly controlled for one factor. This allows for a clearer examination of the basic joint biomechanics.

## 5. Conclusion

This study suggests that the integrity of the ACL plays a protective role in performing medial UKA. However, this protective effect is limited when performing medial UKA. When the knee joint only has varying degrees of ACL injury, even ACL rupture, and the remaining structures of the knee joint are intact with anterior-posterior stability in the knee joint, it should not be considered a contraindication for medial UKA.

## Supporting information

S1 File (ZIP)

## Abbreviations

ACL: Anterior Cruciate Ligament
ACLD: Anterior Cruciate Ligament Deficence
ACLI: Anterior Cruciate Ligament Intact
KOA: Knee Osteoarthritis
MOA: Medial Compartment Osteoarthritis
PCL: Posterior Cruciate Ligament
UKA: Unicompartmental Knee Arthroplasty
